# Effect of radiographic malpositioning on patellar height measurements: implications for diagnosis and surgical planning. A retrospective observational study

**DOI:** 10.1007/s00590-025-04236-1

**Published:** 2025-03-26

**Authors:** Jorge García Cabrera, Sergio Barroso Rosa, Carmen Nieves Hernández Flores

**Affiliations:** 1 University of Las Palmas de Gran Canaria, Palmas de Gran Canaria, Spain; 2 The Orthopaedic Research Institute of Queensland, Townsville, Australia

**Keywords:** Patellar height, Radiography, Positioning, Planning, Diagnostic

## Abstract

**Purpose:**

Patella alta stands as a significant predisposing factor for patellofemoral instability. Patellar heights indexes (PHI) require precise lateral knee radiographic projections for accurate computation. However, within clinical contexts, a notable proportion of radiographic images are taken with certain degrees of excessive rotation and/or tilting. The primary aim of this investigation was to assess the impact of suboptimal radiographic positioning on the determination of patellar height, utilizing the Blackburne-Peel (BP), Caton-Deschamps (CD) and Insall-Salvati (IS) indexes. Secondarily, it was evaluated whether any index is more sensible to suboptimal radiographic positioning, and how inter and intra observer reproducibility are affected.

**Methods:**

Thirty-three patients with strictly lateral radiographs and another one demonstrating a noticeable degree of tilt and/or rotation were included in the study. Four orthopaedic surgeons specialized in knee surgery and four Orthopaedics residents conducted measurements on each radiograph in a randomized sequence at two different time points. A linear mixed-effects model was applied, with the quality of the radiograph (adequate projection vs malrotation, tilt, or both), observer expertise (consultant or resident), and observation time regarded as fixed effects, while consultant and patient were treated as random effects.

**Results:**

Statistically significant differences were obtained between strict lateral and tilted radiographs in the BPI, with an overestimation of up to 0.0937; between strict lateral and malrotated radiographs in the ISI, showing an overestimation of up to 0.0696 and between tilted and/or rotated radiographs in the CDI, with overestimation reaching up to 0.0813. No significant differences were observed between resident and consultant observers in any of the indexes. Good inter-observer consistency was achieved.

**Conclusion:**

This study showed statistically significant differences in the determination of the three PHIs. Although these differences were small, they may predispose to diagnostic errors and inaccurate surgical planning in cases requiring surgical correction. Future studies quantifying the degrees of malrotation and/or inclination may further clarify these findings.

## Introduction

Patellofemoral instability (PFI) is a well-known cause of referral to orthopaedic clinics. Predisposing factors, i.e. troclear dysplasia, patellae alta (PA), insufficiency of medial retinacular structures such as the medial patellofemoral ligament (MPFL) or lower limb malalignment, have been extensively studied over the past decades[1]. Patella alta, this meaning an excessively proximal patellar location regarding the knee joint line, is associated with 30–50% of patellar dislocations [2]. Furthermore, a high riding patella is the only factor capable of causing a low-energy dislocation without associated trochlear dysplasia, making it a crucial instability factor and a pivotal consideration in treatment decisions [3]. Additionally, patellar height is also crucial for managing patients following anterior cruciate ligament reconstruction, high tibial osteotomies and total knee replacements, where a reduced patellar height may lead to postoperative complications, as anterior knee pain [4, 5]. Consequently, evaluating patellar height with precision is essential in diagnosing and treating PFI.

Various patellar height indexes (PHI) attempt to establish simple, reliable and reproducible methods for assessing patellar height based on simple radiology. Most PHI are calculated from lateral knee x-rays obtained in a pure or strict projection. Strictly projected lateral radiographs are characterized by the overlap of the femoral condyles, visible space at the patellofemoral joint (in the absence of advanced osteoarthritis), and slight overlap of the fibular head with the tibia [6]. However, in clinical practice, many radiographs are not taken in a proper projection, but with a certain degree of excessive tilting and/or rotation, which hinders their applicability as tools for diagnosis and/or surgical correction planning.

Currently, there is poor evidence on the effects of these technical imperfections on the assessment of PHI and their potential influence on decision-making for patients with patellofemoral pathology [7]. Errors in PHI measurements may result in incorrect surgical indications, such as unnecessary tibial tubercle osteotomy or failure to address patellofemoral instability adequately. Ensuring accurate radiographic acquisition is therefore crucial to optimizing patient outcomes. The main purpose of this study is to evaluate whether the calculation of patellar height in poorly performed radiographs differs significantly from those obtained in correctly performed ones. Secondary objectives include: 1- evaluating whether rotation or tilting have a greater impact on PHI measurements, 2- determining if any specific index is more susceptible to the effects of poor radiographic technique, and 3- assessing inter- and intra-observer reproducibility. The working hypothesis posits that an inadequate radiographic technique significantly influences PHI measurements due to challenges in locating the necessary anatomical landmarks, predisposing to diagnostic errors and inaccurate surgical planning.

## Methods

This study was designed as a retrospective observational study. Knee radiographs were selected from the already existing in the Radiology Department database; no additional captures were taken for the purpose of this study. All retrieved images were anonymized before analysis; approval from the local Ethics Committee was obtained (CEI/CEIM 2023–446-1).

### Participants

**Fig. 1:**
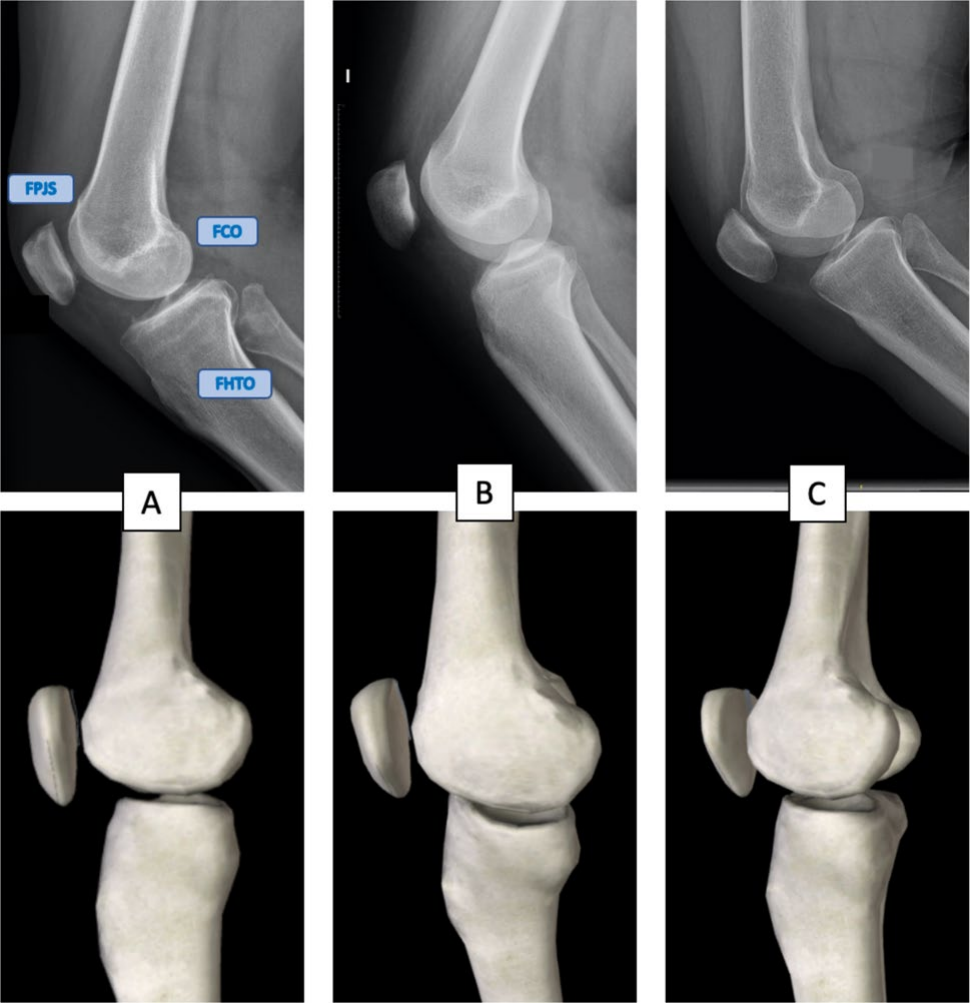
Image A shows a strict lateral projection, as described by Murphy (10): clear femoropatelar joint space (FPJS); overlap of the femoral condyles (FCO) and slight overlap of the fibular head with the proximal tibia (FHTO). Image B shows a tilted lateral projection, with a distortion in the horizontal plane. Image C shows a rotated lateral view, with the distortion occurring in the sagittal plane

A custom-made database was obtained from the Complejo Hospitalario Universitario Insular y Materno Infantil (CHUIMI) Radiology Department database: patients with at least two x-rays from the same knee performed in 2022. 409 patients were reviewed to reach the target sample size. The list was screened to select patients with the following criteria:

- Inclusion criteria:
  - Patients with at least one strictly lateral radiograph and another exhibiting significant malrotation or tilting, on the same knee. Strict lateral projections were defined as those showing overlap of the femoral condyles, femoropatelar joint space, and slight overlap of the fibular head with the tibia, as defined by Murphy (Figure 1) [6]. Image selection was performed by JGC and SBR; the degree of tilting and/or rotation could not be calculated due to the retrospective nature of the study and the lack of angular calibration of the pre-existing images.
- Exclusion criteria:
  - Knee flexion above 70° or below 30°.
  - Patellar fracture.
  - Previous knee surgery.
  - Skeletal immaturity (open tibial/femoral/fibular physis).
  - Gross distal femoral and/or proximal tibial deformities.

### Sample size estimation

A sample size of 33 elements provided a 95% confidence interval and a margin of error of 0.17. Consequently, 33 sets of 2 lateral knee x-rays were obtained.

### Procedures

The 66 radiographs were independently assessed in a randomized sequence by eight observers; 4 senior orthopaedic surgeons specialized in knee surgery and 4 Orthopaedics residents. Measurements were conducted independently on two separate occasions, spaced at least one week apart, in a different image sequence order. All observers were blinded to the patients' information, the results of their colleagues and their own previous calculations. Measurements were obtained using *Horos* (*free and open source code software (FOSS) sponsored by Nimble Co LLC d/b/a Purview in Annapolis, MD USA*) [8].

**Fig. 2:**
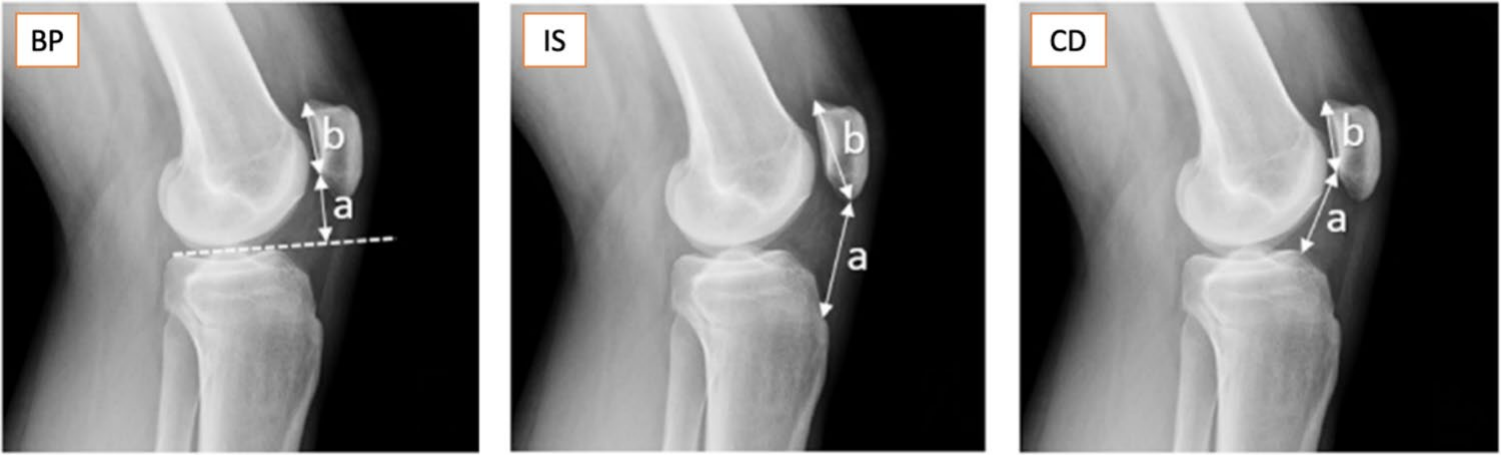
From left to right: BP (Blackbourne-Peel), measured by projecting an anterior intercondylar line and determining the perpendicular distance from this line to the underside of the patellar cartilage (a), which is then divided by the surface area of the patellar cartilage (b). IS (Insall-Salvati), calculated by dividing the length of the patellar tendon (a) by the length of the patella (b). CD (Caton-Deschamps), dividing the distance from the inferior border of the patella to the superior tibial border (a) by the length of the patellar cartilage (b). All three indexes are the result of a/b. BP should be measured with the knee at 30 degrees of flexion or more, IS between 20 and 70 degrees and CD between 10 and 90(1)

Three PHI were calculated, according to the indications in their original publications: Blackburne-Peel ratio (BP), Insall-Salvati index (IS) and Caton-Deschamps ratio (CD). Figure 2 shows a diagram of the indexes and calculation instructions employed by all observers.

### Statistical analysis

A linear mixed-effects model was applied to account for variability among patients and observers [9, 10]. Fixed effects included radiographic quality, observer category, and observation time, while random effects accounted for inter-observer and intra-observer variability. Coefficients (SE), 95% confidence intervals (95% CI), p values were summarized. Statistical significance was set at *p* < 0.05. Data were analysed using R software (*R Core Team (2021). R: A language and environment for statistical computing. R Foundation for Statistical Computing, Vienna, Austria*) [11].

## Results

No statistically significant differences were observed among observations in relation to the level of experience (consultants vs trainees). A summary of the main findings is presented in Table 1. The results obtained for each index are presented below:

**Table 1:** Summary table with mean values, standard deviations, and significant differences

Index	Strict Lateral	Tilting	Malrotation	Combined malpositioning
BPI	0.8434 ± 0.0526	0.9121 ± 0.025 (*p* < 0.001)	0.8571 ± 0.0305 (NS)	0.8954 ± 0.178 (*p* < 0.001)
ISI	1.2359 ± 0.1152	1.2502 ± 0.0221 (NS)	1.1872 ± 0.027 (*p* < 0.001)	1.2462 ± 0.0156 (NS)
CDI	0.9478 ± 0.0557	1.004 ± 0.0248 (*p* < 0.001)	0.9901 ± 0.0305 (*p* = 0.007)	0.9967 ± 0.0178 (*p* < 0.001)

### Blackburne-Peel index

**Fig. 3:**
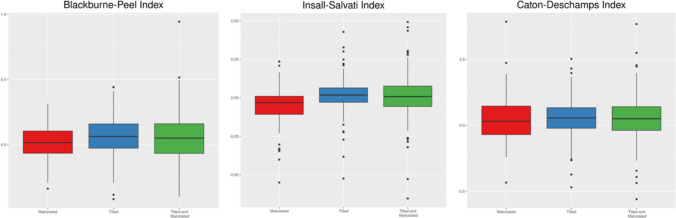
Graphic representation of the linear mixed-effect model results for the analysed PHIs

Statistically significant differences were observed between strictly lateral radiographs and all those with some degree of tilting (Fig. 3). Tilting overestimates the BPI up to 0.0937. Combined tilting and rotation resulted in an index overestimation up to 0.0696. Isolated malrotation did not significantly affect the BPI. Good inter-observer and intra-observer reproducibility were observed for BPI calculations (Table 2).

**Table 2:** Linear mixed-effects model for the BPI

Effect	Coefficient (SE)	CI 95%	*P*
${\sigma }_{P}$	0.1286	(0.1006; 0.1658)	
${\sigma }_{d}$	0.0274	(0.0142; 0.0481)	
$\sigma$	0.1091	(0.1043; 0.1138)	
$\mu$	0.8435 (0.0271)	(0.7909; 0.8961)	< .001
${\alpha }_{I}\left(R\right)$	0.0136 (0.0156)	(− 0.0169; 0.0442)	0.383
${\alpha }_{I}\left(I\right)$	0.0686 (0.0128)	(0.0436; 0.0937)	< .001
${\alpha }_{I}\left(IR\right)$	0.0519 (0.0090)	(0.0341; 0.0696)	< .001
${\beta }_{C}\left(R\right)$	0.0024 (0.0205)	(− 0.0398; 0.0446)	0.911
${\gamma }_{2}$	0.0125 (0.0067)	(− 0.0006; 0.0257)	0.062

### Insall-Salvati index

Excessive rotation resulted in statistically significant differences, up to -0.0757 (Table 3). There were no statistically significant differences found between control radiographs and those with some degree of tilting (Fig. 3). Good inter-observer and intra-observer reproducibility was consistently observed (Table 3).

**Table 3:** Linear mixed-effects model for the ISI . In the model's parameterization, the random effect of patient observation is assumed to be a normally distributed random variable with a mean of 0 and standard deviation ${\sigma }_{p}$, where ${\sigma }_{p}$ represents de the variability among patients. $\mu$ denotes the expected value of the evaluated index. ${\alpha }_{I}\left(I\right)$ represents the difference attributed to a radiograph with only inclination, ${\alpha }_{I}\left(R\right)$ indicates the difference attributed to a radiograph with only rotation and ${\alpha }_{I}\left(IR\right)$ represents the difference attributed to a radiograph with both inclination and rotation simultaneously. ${\beta }_{C}\left(R\right)$ stands for the effect (fixed) of a trainee compared to a senior consultant. ${\gamma }_{2}$ signifies the fixed effect of the observation time, indicating the difference between the first and second observations

Effect	Coefficient (se)	CI 95%	*P*
${\sigma }_{P}$	0.1677	(0.1326; 0.2172)	
${\sigma }_{d}$	0.1031	(0.0595; 0.1661)	
$\sigma$	0.0961	(0.0919; 0.1002)	
$\mu$	1.2359 (0.0595)	(1.1207; 1.3512)	< .001
${\alpha }_{I}\left(R\right)$	− 0.0487 (0.0138)	(− 0.0757; -0.0216)	< .001
${\alpha }_{I}\left(I\right)$	0.0143 (0.0113)	(− 0.0078; 0.0364)	0.206
${\alpha }_{I}\left(IR\right)$	0.0103 (0.0080)	(− 0.0053; 0.0260)	0.196
${\beta }_{C}\left(R\right)$	− 0.0067 (0.0731)	(− 0.1518; 0.1384)	0.930
${\gamma }_{2}$	0.0007 (0.0059)	(− 0.0109; 0.0123)	0.902

### Caton-deschamps index

Statistically significant differences were noted between defective radiographs and strict lateral ones; tilting overestimates the CDI up to 0.0813; rotation up to 0.0728 and both combined effects affected it up to 0.0665 (Fig. 3). Good inter-observer reproducibility was observed; however, intra-observer reproducibility was not satisfactory (Table 4). As a result of the latter, a consistency sub-analysis was performed independently for optimal and suboptimal radiographs (Table 5); good inter-observer reproducibility was achieved regardless of the quality of the radiographic projection, while steady intra-observer reproducibility was achieved with strictly lateral projections, but not with defective ones.

**Table 4:** Linear mixed-effects model for the CDI

Effect	Coefficient	CI 95%	*P*
${\sigma }_{P}$	0.1405	(0.1100; 0.1808)	
${\sigma }_{d}$	0.0266	(0.0137; 0.0470)	
$\sigma$	0.1087	(0.1040; 0.1134)	
$\mu$	0.9478 (0.0286)	(0.8921; 1.0035)	< .001
${\alpha }_{I}\left(R\right)$	0.0423 (0.0156)	(0.0118; 0.0728)	0.007
${\alpha }_{I}\left(I\right)$	0.0562 (0.0127)	(0.0314; 0.0813)	< .001
${\alpha }_{I}\left(IR\right)$	0.0489 (0.0090)	(0.0311; 0.0665)	< .001
${\beta }_{C}\left(R\right)$	0.0099 (0.0200)	(− 0.0313; 0.0510)	0.639
${\gamma }_{2}$	0.0166 (0.0067)	(0.0035; 0.0297)	0.013

**Table 5:** Linear mixed effects model for CDI. X-rays were clustered according to the quality of the radiographic projection

	Correct projections			Suboptimal projections		
effect	coefficient (se)	CI 95%	*P*	coefficient (se)	CI 95%	*P*
${\sigma }_{P}$	0.1450	(0.1135; 0.1871)		0.1496	(0.1169; 0.1929)	
${\sigma }_{d}$	0.0304	(0.0151; 0.0539)		0.0219	(0.0078; 0.0414)	
$\sigma$	0.0971	(0.0913; 0.1035)		0.1036	(0.0974; 0.1104)	
$\mu$	0.9477 (0.0304)	(0.8887; 1.0067)	< .001	0.9976 (0.0293)	(0.9403; 1.0549)	< .001
${\beta }_{C}\left(R\right)$	0.0117 (0.0231)	(− 0.0358; 0.0591)	0.631	0.0080 (0.0179)	(− 0.0291; 0.0452)	0.669
${\gamma }_{2}$	0.0150 (0.0085)	(− 0.0016; 0.0316)	0.076	0.0181 (0.0090)	(0.0004; 0.0358)	0.045

## Discussion

The main finding of this study is that inadequate radiographic projections favour inexact PHI calculations, which may predispose to diagnostic errors and/or inaccurate surgical planning. The main clinical implication would be the assumption that a significant proportion of these decisions factually rely on measurements derived from suboptimal radiographic images; it has been assumed that variations nearing 0.1 in any of the three PHI are deemed clinically relevant, as described by Huddleston et al. [7].

**Fig. 4:**
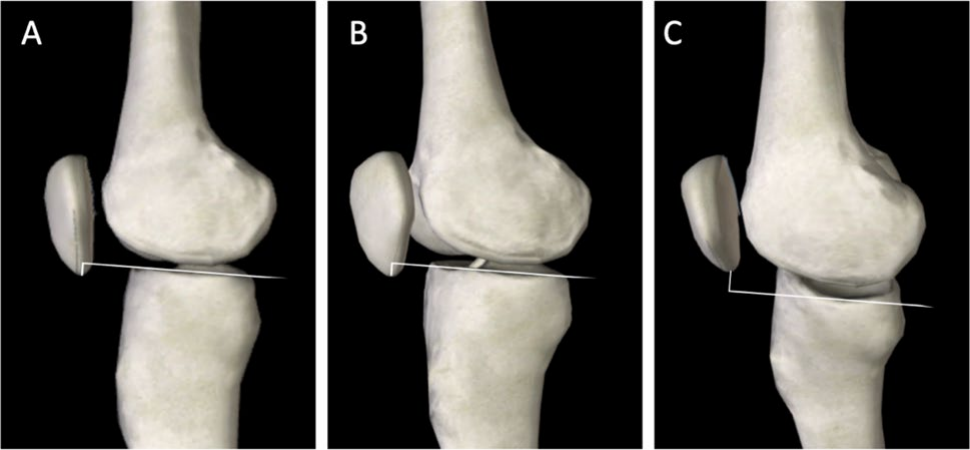
Three-dimensional representation of the effect of rotation and tilting in patellar height variation. Image A represents a strict lateral projection, image B a rotated projection, and Image C a tilted projection. Note how tilting affects patella-joint line distance more than rotation

The BPI has been appointed as one of the most recommendable PHIs, as it provides a direct estimation of patellar position in relation to the joint line [12]. For this index, values are significantly affected by tilting, whereas excessive rotation seems not to cause a relevant impact. This can be easily understood in a three-dimensional model: rotation alters horizontal distances and tilting modifies vertical distances (Fig. 4). A similar effect was found by Bixby et al. for tibial slope calculation [13]. Moreover, when radiographs are not acquired with a perfectly lateral view, the tibial plateaus do not appear parallel in the image, complicating line placement [14] (Fig. 2, left image).

In relation to the Insall-Salvatti index, values showed differences that could be clinically relevant between malrotated and control radiographs, underestimating the patellar height up to 0.0757. These differences could be explained by the increased hassle in locating the anterior tibial tuberosity when the projection is rotated, altering the apparent position of the patellar tendon. No clinically relevant differences were observed between the radiographs with a certain degree of tilting and the controls.

The impact of suboptimal radiographic projection in CDI has been evaluated by Huddleston et al. [7]. They conducted a study involving five cadaveric knees and obtained pure lateral radiographs at 5°, 10°, and 15° of flexion in both clockwise and counter clockwise directions, in the axial and coronal planes. PHI measurements were assessed by three orthopaedic surgeons; statistically and clinically significant results were obtained particularly in cases of counter clockwise malrotation, whereas our study found smaller discrepancies. This discrepancy may be to the differences in methodology, including the degree of malrotation evaluated. Additionally, Huddleston et al. found that greater degrees of malrotation in this plane led to increased errors. Additionally, they proposed that all degrees of malrotation or tilting in both directions have the potential to produce clinically significant changes based on the maximum differences observed in each malrotation state. In the present study, it was observed how CDI is also influenced by both rotation and tilting, resulting in the most sensitive PHI to inadequate radiographic knee positioning. However, the shown discrepancies were smaller compared to those observed by Huddleston et al., which could be attributed to differences in quantification of malrotation and/or tilting degrees, sample size, or number of observers and their level of experience.

In the present study, with multiple observers with diverse experience level, factors of poor radiographic projection and knee flexion have been controlled. Good inter-observer reproducibility was achieved across all indices, regardless of the observers’ expertise. Intra-observer reproducibility is good in ISI and BPI, but not in CDI (Tables 1–3), concurring in part with previous reports [15]. Taking into account that in CDI there was good inter-observer reproducibility, poor intra-observer reproducibility can be explained by already described difficulty in defining the anterior tibial spine, since it often presents a non-acute border [16] and by the additional difficulty due to the effects of malrotation and/or tilting. This is supported by the findings of the CDI sub-analysis in two groups of optimal vs suboptimal radiographs (Table 5).

As seen here, the most frequently utilized in practice PHIs are susceptible to clinically significant alterations due to suboptimal radiographic projection. Currently, advancements in artificial intelligence (AI) are being made to enhance measurement precision. Adleberg et al. developed an AI model that measured the Insall-Salvati index on lateral knee radiographs in a highly consistent manner, with minimal mean distance error [17]. However, it has been previously disclosed that an ideal patellar hight measurement should rely on three-dimensional imaging, rather than on plain x-rays subjected to patient malpositioning [18]. In this regard, several MRI and CT techniques have been described for patellar height calculation, often under quadriceps contraction or even active knee movement [19]. The results of the present study support the convenience of these imaging modalities for a precise assessment of patellar height, a fact that should be taken into account not only for diagnostic purposes but also for surgical planning.

Another critical finding of this study was that most screened images exhibited suboptimal radiographic projections (Fig. 1): it was noted that many excluded patients had between three and ten radiographs lacking strictly lateral views. The analysed database comprise a clinical collection of radiographic studies obtained in a third level European institution; if this situation is extrapolated globally, it could be assumed that a high proportion of clinically used images lack adequate quality. Brunner et al. stated that a combination of altered rotation and flexion in lower limb image acquisition can easily reach clinically relevant alterations of alignment measures [20]. The implications of inadequate radiologic projections have been extensively discussed [21, 22] and healthcare facilities should implement quality protocols to avoid fawlty radiologic examinations [23].

As any other, this study has limitations. A potentially relevant one is the lack of standardized calibration across radiographs, which may introduce variability in measurements. Future studies should aim to quantify malrotation and tilting in degrees for a more precise assessment. Another constraint may be sample size. To achieve a 95% confidence level with a margin of error of 0.05, a total of 384 images would have been necessary; this requirement would have complicated patient selection and collaboration among researchers. However, the achieved confidence level of 95%, with a 0.17 error may be considered excellent for a research of this nature. Among the strengths of this study was the employment of actual clinical practice radiographs, the inclusion of a variety of commonly used PHI, and a large party of researchers performing multiple observations.

## Conclusions

Calculation of patellar heigh indexes is not reliable when inadequate radiological projections are performed. Tilting primarily impacts the BPI, ISI is affected by rotation and the CDI is affected by both rotation and tilting. Deviations in obtained values may be minimal, but do have the potential of resulting in diagnostic or surgical planning errors. Standardized patient positioning and radiography acquisition techniques should be implemented in all health care facilities, ensuring strict lateral projections, minimising measurement variability and thus improving clinical decision-making.

## Data Availability

Research data is available upon reasonable request.

## References

[1] Imhoff FB, Funke V, Muench LN et al (2020) The complexity of bony malalignment in patellofemoral disorders: femoral and tibial torsion, trochlear dysplasia, TT-TG distance, and frontal mechanical axis correlate with each other. Knee Surg Sports Traumatol Arthrosc 28:897–904. 10.1007/S00167-019-05542-Y 31127313 10.1007/s00167-019-05542-y

[2] Muñoz SR, Miranda EA, Iñiguez MC et al (2022) Patellofemoral joint imaging study: State of the art. Revista Chilena de Radiologia 28:12–26

[3] Huntington LS, Webster KE, Devitt BM et al (2019) Factors associated with an increased risk of recurrence after a first-time patellar dislocation: a systematic review and meta-analysis. The American J sports Medicine 48:2552–2562. 10.1177/0363546519888467 10.1177/0363546519888467 31825650

[4] Lum ZC, Saiz AM, Pereira GC, Meehan JP (2020) Patella Baja in Total Knee Arthroplasty. J Am Acad Orthop Surg 28:316–323. 10.5435/JAAOS-D-19-00422 31934927 10.5435/JAAOS-D-19-00422

[5] Otsuki S, Murakami T, Okamoto Y, et al (2018) Risk of patella baja after opening-wedge high tibial osteotomy. J Orthop Surg (Hong Kong) 26: 10.1177/2309499018802484 10.1177/2309499018802484 30295136

[6] Murphy A (2019) Knee (lateral view). Radiopaedia.org. 10.53347/RID-72198

[7] Huddleston HP, Redondo ML, Cregar WM et al (2023) The Effect of Aberrant Rotation on Radiographic Patellar Height Measurement Using Canton-Deschamps Index: A Cadaveric Analysis. Journal of Knee Surgery 36:254–260. 10.1055/s-0041-1731720 34261156 10.1055/s-0041-1731720

[8] Horosproject.org. Horos v3.3.6 [Internet]. Annapolis (MD): Nimble Co LLC d/b/a Purview; [01/2024]. Available from: http://www.horosproject.org/

[9] Bates D, Maechler M, Bolker B, Walker S (2015) Fitting Linear Mixed-Effects Models Using lme4. J Stat Softw 67(1):1–48. 10.18637/jss.v067.i01

[10] Kuznetsova A, Brockhoff PB, Christensen RHB (2017). “lmerTest Package: Tests in Linear Mixed Effects Models.” Journal of Statistical Software, 82(13), 1–26. 10.18637/jss.v082.i1310.18637/jss.v082.i13.

[11] R Core Team (2023). R: A language and environment for statistical computing. R Foundation for Statistical Computing, Vienna, Austria. URL https://www.R-project.org/.

[12] Phillips CL, Silver DAT, Schranz PJ, Mandalia V (2010) The measurement of patellar height: a review of the methods of imaging. J Bone Joint Surg Br 92:1045–1053. 10.1302/0301-620X.92B8.23794 20675745 10.1302/0301-620X.92B8.23794

[13] Bixby EC, Tedesco LJ, Confino JE, Mueller JD, Redler LH (2023) Effects of Malpositioning of the Knee on Radiographic Measurements: The Influence of Adduction, Abduction, and Malrotation on Measured Tibial Slope. Orthopaedic J Sports Medicine. 10.1177/23259671231164670 10.1177/23259671231164670 PMC10280522 37347024

[14] van Duijvenbode D, Stavenuiter M, Burger B et al (2016) The reliability of four widely used patellar height ratios. Int Orthop 40:493–497. 10.1007/s00264-015-2908-2 26178255 10.1007/s00264-015-2908-2

[15] Picken S, Summers H, Al-Dadah O (2022) Inter- and intra-observer reliability of patellar height measurements in patients with and without patellar instability on plain radiographs and magnetic resonance imaging. Skeletal Radiol 51:1201–1214. 10.1007/s00256-021-03937-y 34718849 10.1007/s00256-021-03937-y

[16] Grelsamer RP, Meadows S (1992) The modified Insall-Salvati ratio for assessment of patellar height. Clinical Orthopaedics and Related Res 282:170–176 1516309

[17] Adleberg J, Benitez CL, Primiano N, Patel A, Mogel D, Kalra R, Ngeow J (2024) Fully Automated Measurement of the Insall-Salvati Ratio with Artificial Intelligence. J Imaging Informatics in Medicine 37(2):601–610 10.1007/s10278-023-00955-1 PMC11031523 38343226

[18] Muhle C, Brossmann J, Heller M (1999) Kinematic CT and MR imaging of the patellofemoral joint. Eur Radiol 9:508–518. 10.1007/S003300050702 10087126 10.1007/s003300050702

[19] Rosa SB, Ewen PM, Doma K et al (2019) Dynamic Evaluation of Patellofemoral Instability: A Clinical Reality or Just a Research Field? A Literature review. Orthop Surg 11:932–942. 10.1111/OS.12549 31797563 10.1111/os.12549 PMC6904628

[20] Brunner J, Jörgens M, Weigert M et al (2023) Significant changes in lower limb alignment due to flexion and rotation—a systematic 3D simulation of radiographic measurements. Knee Surg Sports Traumatol Arthrosc 31:1483. 10.1007/S00167-022-07302-X 36595052 10.1007/s00167-022-07302-x PMC10050026

[21] Barroso Rosa S, Felipe Peña M, Díez Izquierdo M, Grant A (2022) We should not accept inappropriate radiologic views. Radiography 28:574–575. 10.1016/j.radi.2022.02.009 35279400 10.1016/j.radi.2022.02.009

[22] Olivetti L, Fileni A, De Stefano F et al (2008) The legal implications of error in radiology. Radiol Med 113:599–608. 10.1007/S11547-008-0279-0 18536873 10.1007/s11547-008-0279-0

[23] ACR-SPR-SSR Practice parameter for the performance of radiography of the extremities

